# Supplementary material for: Insights into the Composition of a Co-Culture of 10 Probiotic Strains (OMNi BiOTiC® AAD10) and Effects of Its Postbiotic Culture Supernatant
Source: Nutrients. 2022 Mar 11;14(6):1194. doi: 10.3390/nu14061194 (PMC8952306; doi:10.3390/nu14061194)
Supplement: Supplementary file 1 [file nutrients-14-01194-s001.zip › nutrients-1609721-supplementary.pdf]

Supplementary Materials

Supplementary methods

Bacterial Culture Information

Bacteria for resistance testing were obtained from Aurosan GmbH, Essen, Germany. Clostridium difficile (ATCC 700057), Listeria monocytogenes (ATCC 15313), Escherichia coli (ATCC 25922), Enterococcus faecium (ATCC 27270), Staphylococcus (S.) aureus (ATCC 29213), Staphylococcus (S.) epidermidis (ATCC 12228), Streptococcus (Str.) agalactiae (ATCC 13813), Pseudomonas (P.) aeruginosa (ATCC 27853), and Propionibacterium (Pr.) acnes (ATCC 6919) were chosen for bacterial resistance testing. After thawing, bacteria were pre-cultured as broth cultures. Aerobic bacteria were cultivated for 24 hours at 37 °C and 120 rpm and anaerobic bacteria for 48 h at 37 °C and 360 rpm. Of each pre-culture, 300 µl were then spread on 15 cm diameter plates with either tryptic soy agar (30 g/l TSB, Fluka Analytical, no T8907-500G, Honeywell, Charlotte, NC, USA; 15 g/l Agar-Agar, Kobe I, no5210-2, Carl Roth GmbH, Karlsruhe, Germany; TSA) for E. coli, S. aureus and P. aeruginosa, TSA with 5 % freshly harvested sheep blood for C. difficile, Str. agalactiae and Pr. acnes, brain heart infusion agar (Brain-Heart-Infusion, no X915.1, 52 g/l, Carl Roth GmbH, Karlsruhe, Germany; 15 g/l Agar-Agar Kobe I, no5210-2, Carl Roth GmbH, Karlsruhe, Germany) for E. faecium and L. monocytogenes or nutrient agar (1 g/l beef extract powder, no B4888-50G, Sigma-Aldrich Handels GmbH, Vienna, Austria; 5 g/l peptone, no P0431-250G, Sigma-Aldrich, Handels GmbH, Vienna, Austria;5 g/l NaCl and 15 or 50 g/l Agar-Agar) for S. epidermidis. Anaerobic bacteria (C. difficile and Pr. acnes) were cultured for 48 hours in boxes with oxid Anaerogen 2.5 l (Thermo Fisher Scientific, Waldham, MA, USA). The remaining aerobic bacteria grew for 24 hours until a dense bacterial lawn was achieved. Candida albicans thankfully provided by PD Dr. Angela Horvath was also chosen for resistance testing as representative of fungi. After pre-cultivation on broth culture, brain heart infusion agar plates (Brain-Heart-Infusion, no X915.1, 52 g/l, Carl Roth GmbH, Karlsruhe, Germany; 15 g/l Agar-Agar Kobe I, no5210-2, Carl Roth GmbH, Karlsruhe, Germany) were used to cultivate C. albicans at 25 °C for 24 hours and an agitation of 120 rpm for both culture steps until a dense fungal lawn was achieved.

Each microorganism was cultured five times on different plates providing the preferred medium for optimal growth for each bacterium. Using a stencil, 9 disks for resistance testing (BD Sensi-Disc^{tm}, Becton, Dickinson and Company, Franklin Lakes, New Jersey, USA) were placed on each culture plate using a prepared scheme. For resistance testing, supernatant was slowly thawed to 4°C in the fridge and then brought to room temperature shortly before use. Each disk was either treated with 20 µl of pure supernatant, a 1:2 or 1:4 dilution of the supernatant, cooked supernatant (100 °C for 30 min, no rotation, Thermomixer, HLC, Germany), buffered supernatant (to pH 7 with 1 n NaOH), supernatant mixed with 1 n HCl (1:1) or supernatant treated with 1 mg/ml Proteinase K (Carl Roth, Germany). Culture medium of the production steps (MRS) served as negative control and antibiotics as positive control. Either vancomycin (Vancomycin Hikma® 500mg, Hikma Pharma, Planegg, Germany; 0.03 mg/disk); for C. difficile, Str. agalactiae, E. faecium, S. epidermidis, L. monocytogenes and S. aureus) or Piperacillin/Tacobactam (PIPeracillin/TAZobactam Kabi 4 g/0.5 g, Fresenius Kabi, Graz, Austria; 0.1 mg/disk) for E. coli, P. aeruginosa and Pr. acnes were used. Plates were then incubated at 37 °C for 24 hours in case of aerobic and for 48 hours in case of anaerobic bacteria.

Thereafter, plates were photographed and inhibition zones were determined with ImageJ 2.0.0-rc-69/1.52p (ImageJ open source image processing software, http://imagej.net/Contributors).

Metabolomics Sample Preparation, Quality Control and Measurement

Samples were thawed from -80 °C in water-ice baths and then vortexed. Thereafter, 100 µl of sample were transferred to 0.5 ml LoBind Eppendorf vials. 400 µl of pre-cooled MeOH (Honeywell, HPLC Grade) were added to each sample, the mixture was vortexed and stored at -80 °C overnight. Samples were then thawed at 4 °C and centrifuged at 14,000 g for 10 minutes at 4 °C. The supernatant was transferred into 2 ml protein LoBind Eppendorf vials and concentrated with N2 until dry. After adding 100 µl of 30 % MeOH they were vortexed for 30 seconds and again centrifuged at 14,000 g for 5 minutes at 4 °C. The supernatant was then aliquoted into autosampler vials (glas inlet; 40 µl each).

Sample analysis was done using a UHPLC Vanquish coupled to a Q-Exactive mass spectrometer (Thermo Fisher Scientific) equipped with a NH2-Luna HILIC analytical column and crudcatcher. Injection volume was 10 µl and metabolite separation was achieved with a 37-min gradient:aqueous acetonitrile solution [(5 % acetonitrile v/v), 20 mM ammonium acetate, 20 mM ammonium hydroxide, pH 9.45] was used as eluent A (LMA) and acetonitrile as eluent B (LMB). Samples were measured in a stratified randomized sequence. Electrospray ionization (ESI) was used for negative and positive ionization and masses between 70 and 1050 m/z were detected.
The Metabolika Database of the Compound Discover 3.1® Software (Thermo Fisher Scientific) was used to further enhance the pathway annotation of metabolites of interest.

After sample analysis, raw data was converted into mzXML files by msConvert (ProteoWizard Toolkit v3.0.5), and known metabolites were searched for with the tool PeakScout [developed by Joanneum Research Graz, Austria] using a reference list containing accurate mass and retention times acquired via reference substances. Quality control was conducted as described in Vogel et al. using Tibco Spotfire V7.5.0.

After quality controls, 122 metabolites were identified, 98 showing very high quality (suitable for multi- and univariate statistics – MVA and UVA, respectively) and 24 high quality suitable for UVA only. For further analysis, only 98 metabolites with very high quality were used.Technical variability was excellent, with a median relative standard deviation (RSD) of 4.4 % for MVA_UVA Metabolites in Quality Control Samples (QC). RSD in groups represents biological variability from samples and technical variability (e.g. sample collection, storage, extraction, measurement). Intra-group variability RSD lies between 38.0 % for samples.

A subsequent assessment using t-Distributed Stochastic Neighbor Embedding (t-SNE) with all quality-controlled metabolites confirmed the good quality of the measurements, showing how (1) pooled samples for quality control (QCs), (2) our internal technical quality control standard mixture (UM, as described in Vogel et al. DOI: doi:10.1016/j.jid.2019.06.124), and (3) blanks (30% MeOH- 70% MilliQ water) formed clusters. In addition, samples also formed a cluster and most replicates were virtually overlapping (v3.31.0, Orange Data Mining Toolbox, Lubljana, Slovenia).

DNA Isolation

For total DNA isolation, fecal samples were isolated with the Magna Pure LC DNA III Isolation Kit (Bacteria, Fungi) (Roche, Mannheim, Germany). Briefly, one stool pellet was mixed with 500 µl PBS and 250 µl bacterial lysis buffer. Samples were homogenized and bead beaten in Magna Lyzer Green bead Tubes (Roche, Mannheim, Germany) in a Magna Lyzer instrument (Roche, Mannheim, Germany) at 6,500 rpm for 30 seconds two times. Followed by enzymatic lysis with 25 µl lysozyme (100 ng/ml, 37 °C for 30 minutes) and 43.4 µl proteinase K (20 mg/ml, 65 °C for 1 hour) samples were heat inactivated at 95 °C for 10 minutes and total DNA was purified in a MagnaPure LC instrument according to manufacturer´s instructions. Total DNA was eluted in 100 µl elution buffer and stored at -20°C until analysis.

16S Based Microbiome Analysis

For 16S PCR, 2 µl of total DNA were used as template in a 25 µl PCR reaction with the FastStart™ High Fidelity PCR-System (Sigma, Darmstadt, Germany) according to manufacturer’s instructions and the target specific primers 515F (5′-GTGYCAGCMGCCGCGGTAA-3′) and 806R (5′-GGACTACNVGGGTWTCTAAT-3′) for 30 cycles in triplicates. Triplicates were pooled, normalized, indexed and purified. The final pool was sequenced on an Illumina MiSeq desktop sequencer at 9 pM and v 3 600 cycles chemistry. FastQ raw files were used for data analysis.

A total of 2,479,083 MiSeq paired end FASTQ reads were used for further analysis. The DADA2 pipeline for modeling and correcting Illumina-sequenced amplicon errors for quality-filtering was used with standard settings for denoising, dereplicating, merging and check for chimeras as implemented in QIIME2 2018.4 microbiome bioinformatics platform. QIIME2 was integrated in an own non-public instance of Galaxy (MedBioNode https://galaxy.medunigraz.at). Taxonomic assignment of the DADA2 representative sequences was provided with the QIIME2 sklearn-based classifier against SILVA rRNA database release 132 at 99% identity. Absolute counts from OTU table on genus level were used to assess abundance changes.

To interpret and compare taxonomic information 16S rRNA data was transferred to the Calypso online software (Calypso 8.84®, accessible through http://cgenome.net/wiki/index.php/Calypso). Samples were rarefied to a read depth of 14,420 (supernatant cultured for 48 hours) or 14,111 (supernatant cultured for 196 hours). Alpha diversity was calculated using Chao1 estimator, Inversed Simpson and Shannon index. Beta diversity was examined using a redundancy analysis (RDA) and colored principal component analysis plots (PCoA) based on Bray-Curtis dissimilarity score. P-values were adjusted for multiple testing by FDR. The identification of discriminating taxa between the groups was performed with a linear discriminant effect size (LEfSe) analysis. Differentially abundant taxa identified by LEfSe analysis were considered relevant if the differences between groups could be verified by ANOVA ($p<0.1$).

Figure S1. Heatmap of Gene Expressions of *Lactobacillus acidophilus*.

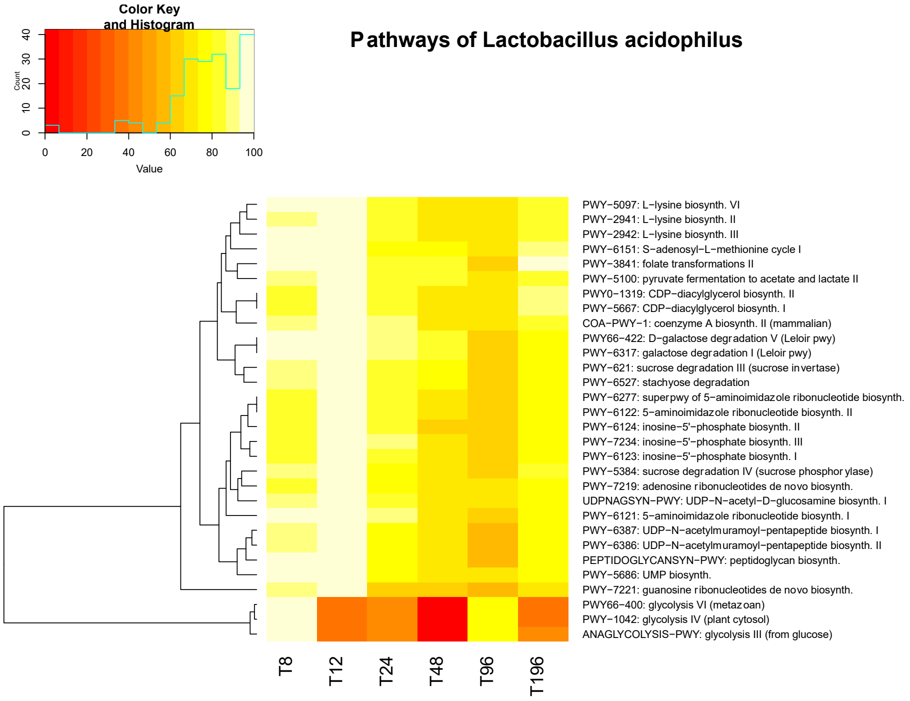

Figure S2. Heatmap of Gene Expressions of *Bifidobacterium longum and lactis*.

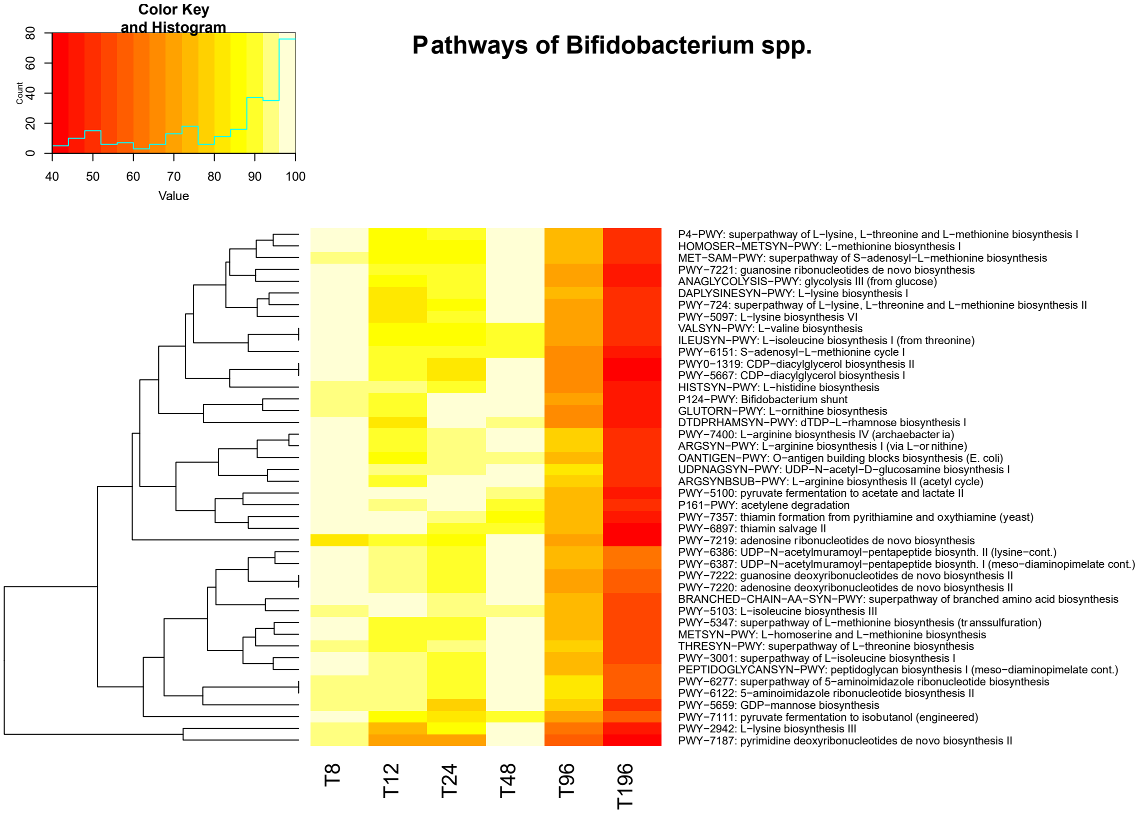

Figure S3. Heatmap of Gene Expressions of *Bifidobacterium bifidum*.

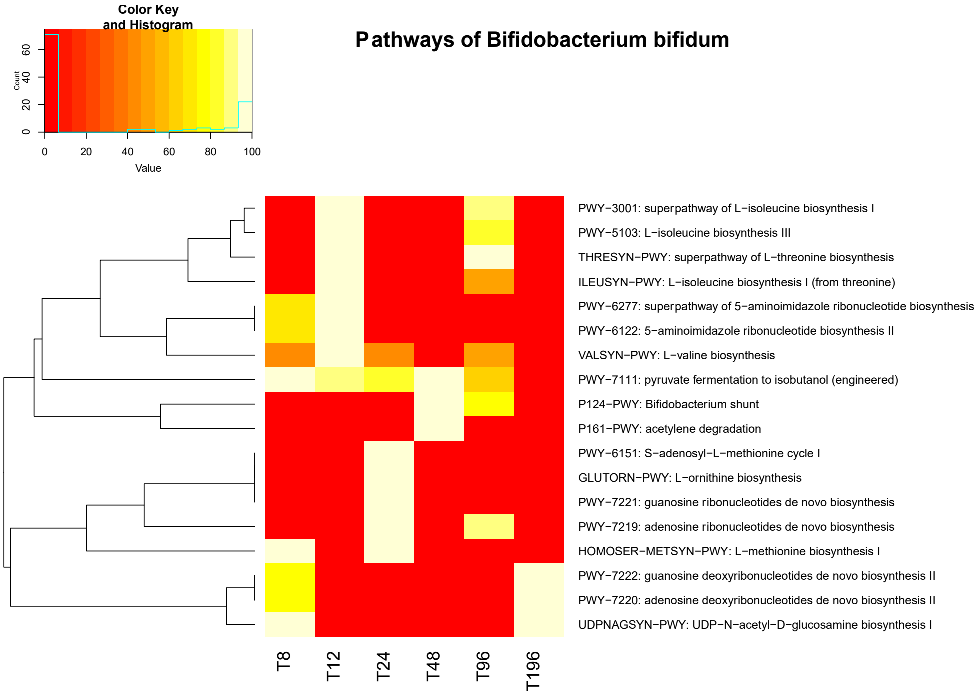

Figure S4. Heatmap of Gene Expressions of *Enterococcus faecium*.

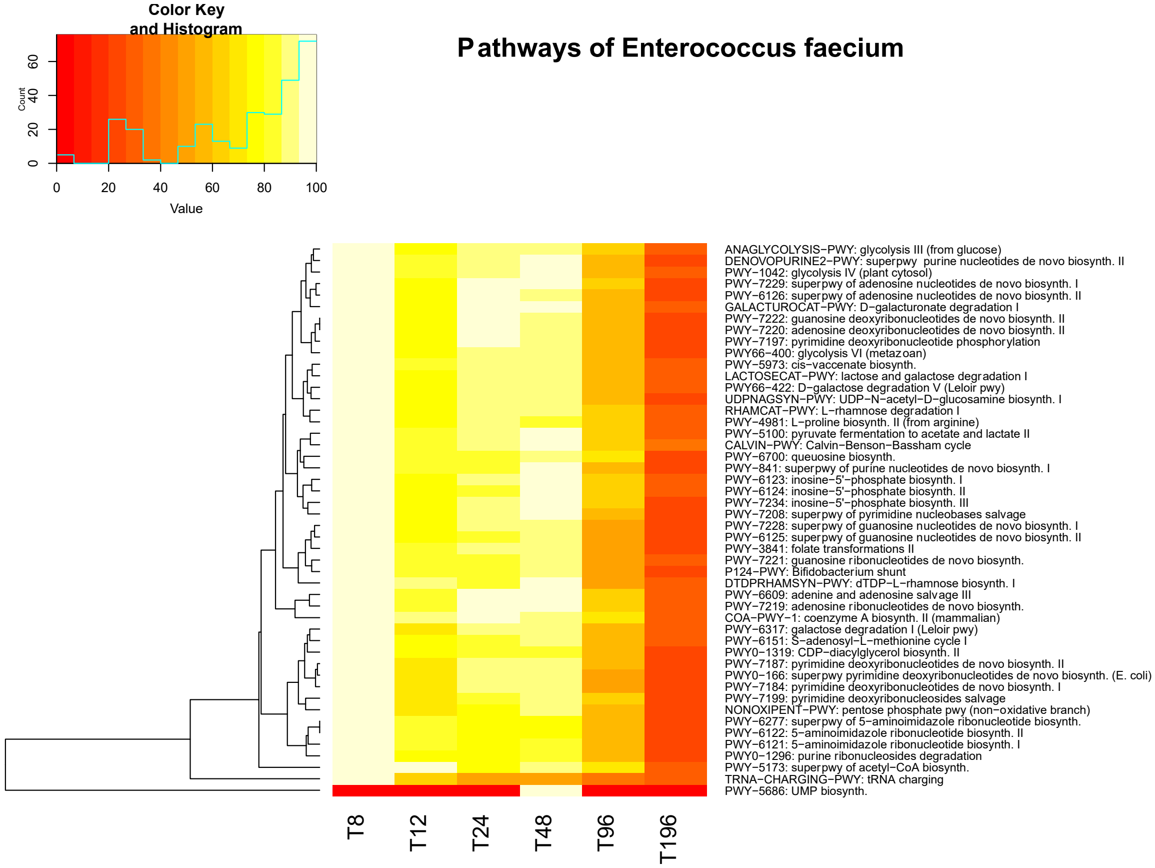

Figure S5. Heatmaps of Gene Expressions of *Lactobacillus paracasei*.

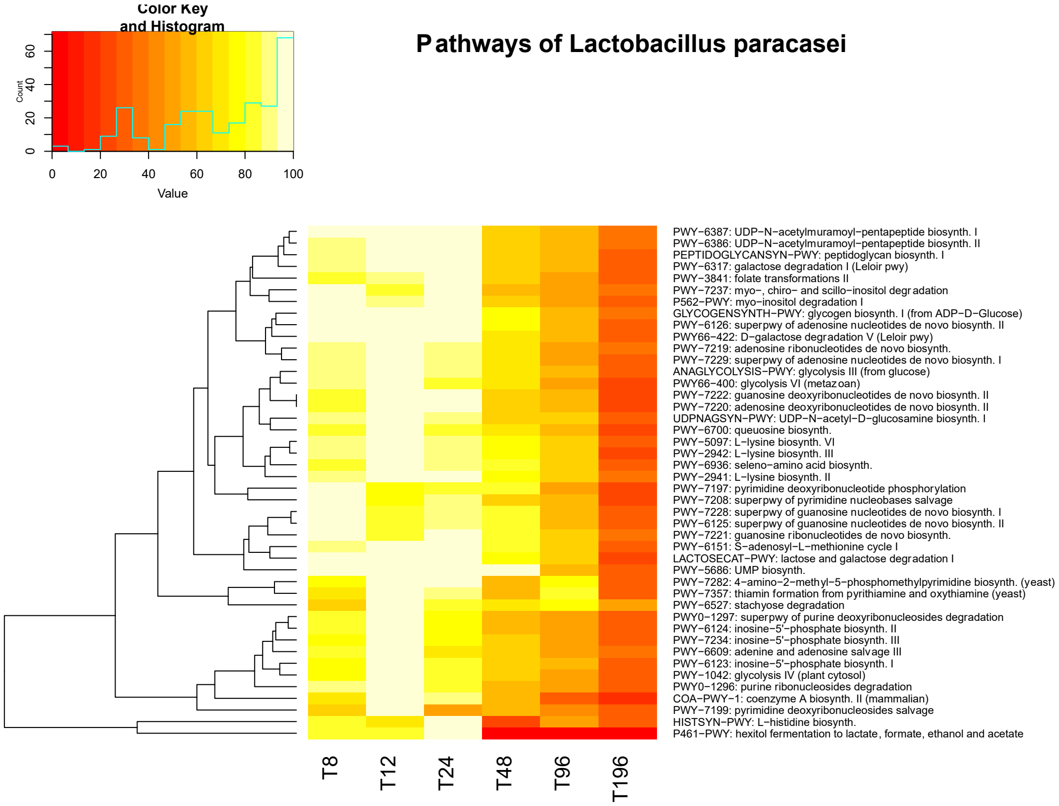

Figure S6. Heatmaps of Gene Expressions of *Lactobacillus plantarum*.

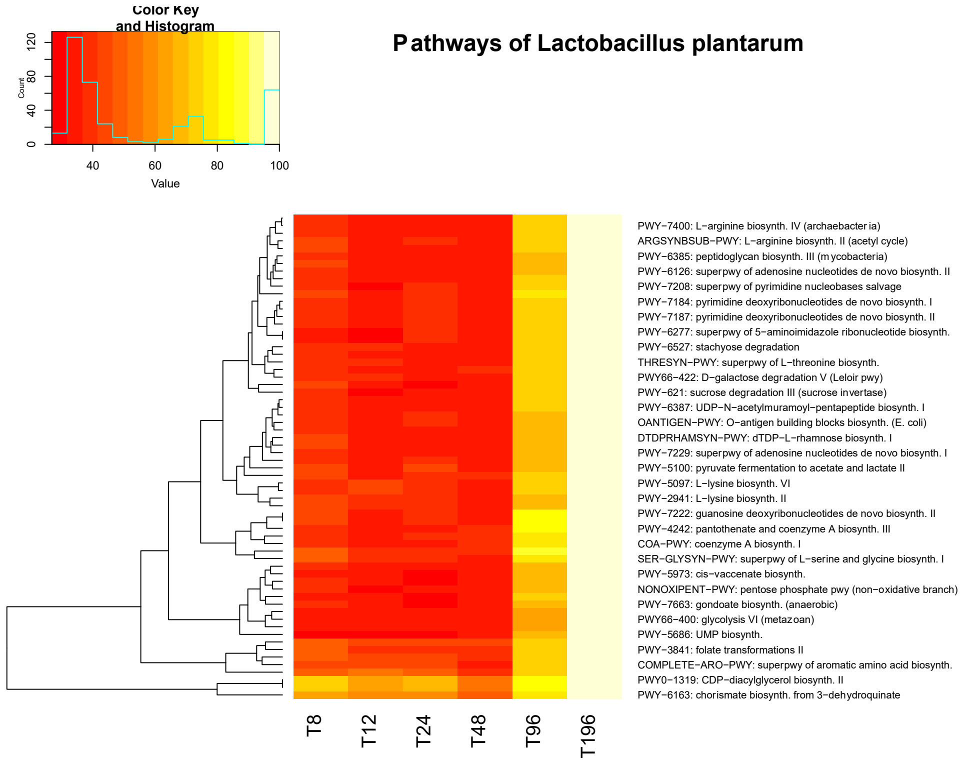

Figure S7. Heatmaps of Gene Expressions of *Lactobacillus rhamnosus*.

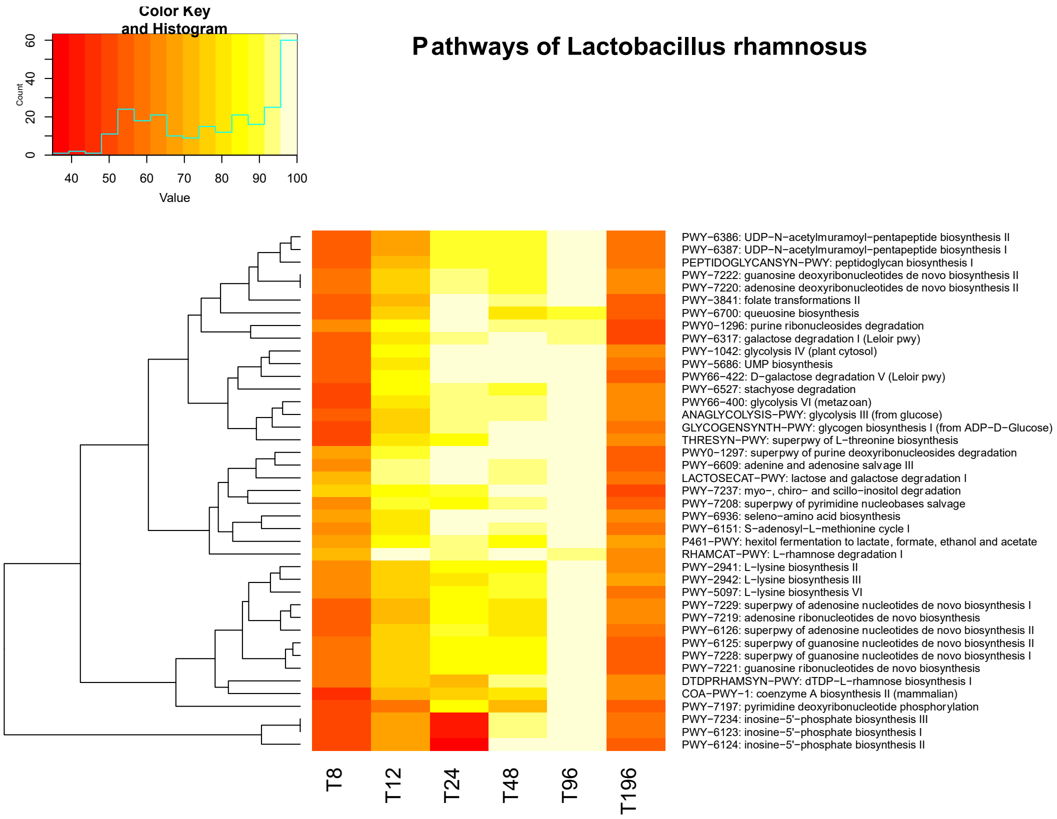

Figure S8. Heatmaps of Gene Expressions of *Lactobacillus salivarius*.

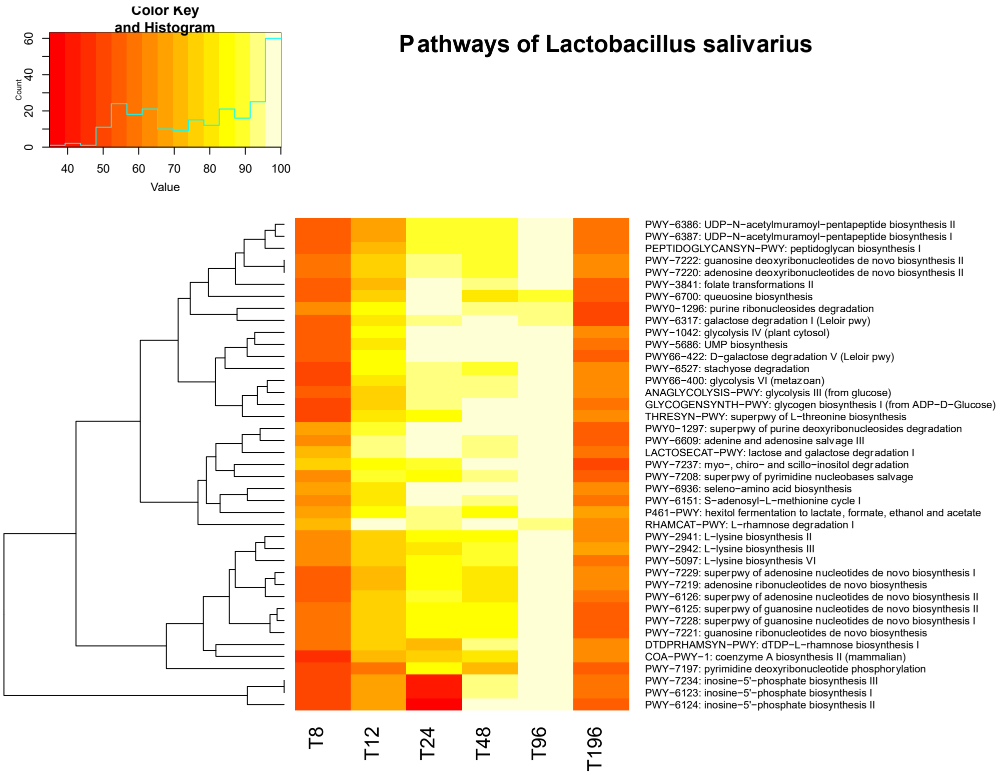

Figure S9. Pie charts of relative abundances of bacteria in murine fecal samples at phylum and family level.

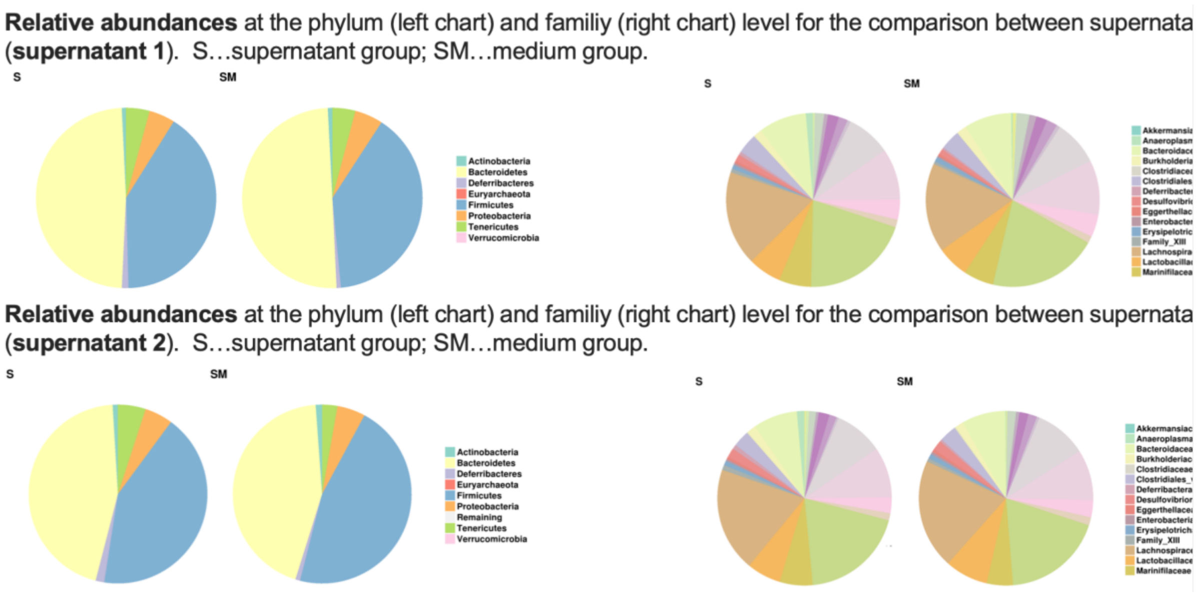

Table S1: Pathways attributed to substances altered over time in the metabolomic analysis

Top 5 Positive Correlations

Fumaric acid	C00122	444972	Citrate cycle (TCA cycle); Oxidative phosphorylation; Arginine biosynthesis; Alanine, aspartate and glutamate metabolism; Tyrosine metabolism; Phenylalanine metabolism; Pyruvate metabolism; Styrene degradation; Butanoate metabolism; Carbon fixation pathways in prokaryotes; Nicotinate and nicotinamide metabolism	3-phenylpropanoate degradation; L-arginine biosynthesis I (via L-ornithine); L-glutamate degradation VIII (to propanoate); Superpathway of adenosine nucleotides de novo biosynthesis I; Superpathway of adenosine nucleotides de novo biosynthesis II; Superpathway of anaerobic energy metabolism (invertebrates); Superpathway of arginine and polyamine biosynthesis; Superpathway of purine nucleotides de novo biosynthesis I; Superpathway of purine nucleotides de novo biosynthesis II
Malic acid	C00149	222656	Citrate cycle (TCA cycle); Pyruvate metabolism; Glyoxylate and dicarboxylate metabolism; Methane metabolism; Carbon fixation pathways in prokaryotes	Anaerobic energy metabolism (invertebrates, mitochondrial); L-glutamate degradation VIII (to propanoate); Superpathway of anaerobic energy metabolism (invertebrates)
Aspartic acid	C00049	5960	Arginine biosynthesis; Alanine, aspartate and glutamate metabolism;	Superpathway of pyrimidine deoxyribonucleotides de novo biosynthesis;

			Glycine, serine and threonine metabolism; Monobactam biosynthesis; Cysteine and methionine metabolism; Lysine biosynthesis; Histidine metabolism; beta-Alanine metabolism; D-Amino acid metabolism; Nicotinate and nicotinamide metabolism; Pantothenate and CoA biosynthesis; ABC transporter; Bacterial chemotaxis	Superpathway of histidine, purine, and pyrimidine biosynthesis; Superpathway of indole-3-acetate conjugate biosynthesis; Superpathway of purine nucleotide salvage; Superpathway of arginine and polyamine biosynthesis; L-arginine biosynthesis I (via L-ornithine); Aspartate superpathway; Superpathways of coenzyme A biosynthesis I; Superpathway of pyrimidine ribonucleotides de novo biosynthesis; Superpathway of coenzyme A biosynthesis I; Methanobacterium thermoautotrophicum biosynthetic metabolism; Superpathway of L-isoleucine biosynthesis I; Superpathway of S-adenosyl-L-methionine biosynthesis; Superpathway of L-methionine biosynthesis (transsulfuration); L-homoserine and L-methionine biosynthesis; Peptidoglycan biosynthesis IV (Enterococcus faecium); Peptidoglycan biosynthesis V (ss-lactam resistance); Superpathway of nicotine biosynthesis; Superpathway of anaerobic energy metabolism (invertebrates); Superpathway of purine nucleotides de novo biosynthesis II; Superpathway of L-methionine biosynthesis (by sulfhydrylation); Superpathway of L-asparagine biosynthesis; Superpathway of L-citrulline metabolism; Superpathway of purine nucleotides de novo biosynthesis I; Superpathway of polyamine biosynthesis III; Superpathway of L-threonine biosynthesis; Superpathway of L-lysine, L-threonine and L-methionine biosynthesis II; Superpathway of L-aspartate and L-asparagine biosynthesis; Superpathway of adenosine nucleotides de novo biosynthesis II; Superpathway of adenosine nucleotides de novo biosynthesis I
Orotidine	C01103	92751	Pyrimidine metabolism; Biosynthesis of cofactors; Urea cycle	Orotidine-5’-Phosphate- Superpathway of pyrimidine ribonucletides de novo biosynthesis; Superpathway of pyrimidine deoxyrubonucleotides de novo biosynthesis;
Cytidine monophosphate	C00055	6131	Pyrimidine metabolism	Methanobacterium thermoautotrophicum biosynthetic metabolism; Superpathway of pyrimidine ribonucleosides

				salvage; Superpathway of phospholipid biosynthesis II (plants); Superpathway of lipopolysaccharide biosynthesis; Superpathway of coenzyme A biosynthesis I; Superpathways of coenzyme A biosynthesis I; Superpathway of (Kdo)2-lipid A biosynthesis; Superpathway of choline biosynthesis; Superpathway of inositol phosphate compounds; Archaetidylinositol biosynthesis; Superpathway of ergosterol biosynthesis II; Superpathway of geranylgeranyl diphosphate biosynthesis II (via MEP); Superpathway of phosphatidylcholine biosynthesis; Taxadiene biosynthesis (engineered); Superpathway of phospholipid biosynthesis I (bacteria); Isoprene biosynthesis I; Archaetidylserine and archaetidylethanolamine biosynthesis; Phosphatidylglycerol biosynthesis I (plastidic); Phosphatidylglycerol biosynthesis II (non-plastidic); Cardiolipin and phosphatidylethanolamine biosynthesis (Xanthomonas); Kdo transfer to lipid IVA III (Chlamydia)

Top 5 Negative Correlations

Phosphoserine	C01005	68841	Glycine, serine and threonine metabolism; Cysteine and methionine metabolism; Methane metabolism; Aminoacyl-tRNA biosynthesis;	Superpathway of sulfate assimilation and cysteine biosynthesis; Superpathway of L-serine and glycine biosynthesis I
Creatine	C00300	586	Glycine, serine and threonine metabolism; Arginine and proline metabolism	N.A.
Pantothenic acid	C00864	6613	beta-Alanine metabolism; Pantothenate and CoA biosynthesis;	Superpathway of coenzyme A biosynthesis I; Superpathway of coenzyme A biosynthesis II (plants); Superpathway of coenzyme A biosynthesis III (mammals); Superpathways of coenzyme A biosynthesis I; Superpathways of coenzyme A biosynthesis III (mammals)
Tryptophan	C00806	1148	Kynurenine metabolism; Protein biosynthesis	NAD biosynthesis II (from tryptophan); Superpathway of

				aromatic amino acid biosynthesis; Superpathway of chorismate metabolism; Superpathway of L-tryptophan biosynthesis; Superpathway of NAD biosynthesis in eukaryotes; Superpathway of roquefortine, meleagrin and neoxaline biosynthesis; L-tryptophan degradation XI (mammalian, via kynurenine); Superpathway of aromatic compound degradation via 3-oxoadipate; Superpathway of fumitremorgin biosynthesis; Superpathway of ergotamine biosynthesis; Superpathway of aromatic compound degradation via 2-oxopent-4-enoate; L-tryptophan degradation III (eukaryotic); L-tryptophan degradation IX; L-tryptophan degradation XII (Geobacillus)
3-Methyl-2-oxovaleric acid	C00671	439286	Valine, leucine and isoleucine degradation/ biosynthesis; Glucosinolate biosynthesis	Superpathway of branched chain amino acid biosynthesis; Superpathway of L-isoleucine biosynthesis I; Superpathway of L-threonine metabolism
